# Partial rupture of anterior cruciate ligament: preliminary experience of selective reconstruction

**DOI:** 10.1186/s10195-020-0544-0

**Published:** 2020-03-28

**Authors:** Christian Carulli, Matteo Innocenti, Giuliana Roselli, Luigi Sirleo, Fabrizio Matassi, Massimo Innocenti

**Affiliations:** 1 Orthopaedic Clinic CTO, University of Florence, Largo Palagi 1, 50139 Florence, Italy; 2 Department of Radiology at Orthopaedic Clinic CTO, University of Florence, Florence, Italy

**Keywords:** Knee, Anterior cruciate ligament, Partial lesions, Partial reconstruction

## Abstract

**Background:**

Partial lesions of the anterior cruciate ligament (ACL) are more common than is generally thought, accounting for about 10–12% of ACL injuries. Selective reconstruction may be considered as an option in isolated bundle rupture. The purpose of this study is to evaluate both subjective and objective clinical results, as well as functional recovery time, after selective arthroscopic single-bundle reconstruction in a consecutive series of patients affected by partial ACL rupture.

**Materials and methods:**

Thirty-six patients undergoing selective reconstruction of a single ACL bundle were retrospectively evaluated from a series of 354 ACL reconstructions performed over a 3-year period. Although the suspicion of partial lesions was present at clinical and magnetic resonance imaging (MRI) evaluation, final diagnosis was obtained during arthroscopy. All patients were operated using the same technique and type of fixation, and undergoing the same functional recovery protocol.

**Results:**

Mean follow-up was 64 months (48–84 months). All patients but one achieved good functional recovery and returned to their sports within a mean period of 6.1 months. A single patient complained of postoperative instability 1 year after the index operation and needed further surgery. No complications were recorded.

**Conclusions:**

Selective reconstruction of partial ACL injury is a method to bear in mind because it offers quick functional recovery. Specific technical and diagnostic steps should be performed and discussed with patients preoperatively.

**Level of evidence:**

Level 4, retrospective study.

## Background

The anterior cruciate ligament (ACL) is made up of two bundles, i.e., anteromedial (AM) and posterolateral (PL), characterized by different functions during tibial rotation and translation throughout the range of motion (ROM). The posterolateral bundle is mostly tight in extension, limiting tibial rotation, while the anteromedial bundle is rather tight in flexion, limiting anteroposterior translation of the knee [2, 9]. ACL injury is common during sport activity and arises most frequently from a noncontact pivoting injury, typically a change of direction or deceleration maneuver [12, 19, 39]. Depending on the position of the knee (flexion, rotation, and adduction/abduction) and the energy transfered to the knee during the sprain, an injury may affect one or both bundles. Isolated injuries account for nearly half of knee ligament injuries: the annual incidence in the USA is about 200,000, with at least 100,000 undergoing arthroscopic reconstruction [17]. The great majority of ACL tears are complete, while partial ACL tears account for 5–27% of all ruptures [1, 8]. Unfortunately, there is no consensus regarding the definition of partial ACL tear yet. For Noyes et al. and Hong et al., it is based on the percentage of remnant fibers [18, 25]; according to Crain et al. and Sonnerycottet et al., it depends on the arthroscopic evaluation [10, 35]; and for the American Medical Association, on the clinical assessment [29]; finally, for DeFranco and Bach, it is multifactorial [12].

Such tears are mostly missed on standard clinical examination, frequently presenting a positive Lachman test; but they show a firm end-point, less than 5 mm differential laxity, and negative or grade I pivot shift test (even if the latter is known to be difficult to assess, particularly in an acute setting) [21]. MRI may not clarify the pattern when not performed by multiple and dedicated slices for ACL evaluation or with a scanner less powerful than 3.0 T [11, 14]. Thus, definitive diagnosis is often obtained by direct evaluation of the ACL during arthroscopic surgery.

Better knowledge of the anatomy, biomechanics, and natural history of partial ACL tears has led to a modern surgical approach. Preserving the residual noninjured bundle while performing selective reconstruction of the torn bundle may be a surgical alternative potentially associated with biomechanical, vascular, and proprioceptive advantages for the patient. Indeed, ACL remnants may add biomechanical strength during the immediate postoperative period, when graft strength depends primarily on the fixation device [3, 24]. The residual portion of the ACL may also maintain its blood supply, supporting the healing process of the graft [13, 15]. Moreover, the proprioceptive properties of the residual bundle may improve the final proprioception of the graft [4, 31, 32]. Furthermore, the intact bundle plays a technical role, helping in proper placement of the bone tunnels and serving as a guide for orientation [34]. Finally, all these aspects may allow accelerated rehabilitation and faster return to sport.

The purpose of this study is to evaluate both the subjective and objective clinical results as well as the functional recovery time after selective arthroscopic single-bundle reconstruction in a consecutive series of patients affected by partial ACL rupture.

## Materials and methods

### Patients and selection criteria

The medical records of 36 patients undergoing selective reconstruction of a single ACL bundle from a series of 354 of ACL reconstructions performed at our institution over a 3-year period (January 2014 to November 2016) were retrospectively evaluated. The demographic data and patients’ characteristics are listed in Table 1.

**Table 1:** Demographics and characteristics of patients

Sex	30 males, 6 females
Mean (range) age	26.4 years (15–37 years)
Interval (range) between injury and surgery	2.6 months (0.2–5 months)
Cause of ACL tear	95% sport injuries (76% pivoting/noncontact trauma)
Type of activity	Football (79%), skiing (5%), volleyball (16%)
Dominant side affected	24

Inclusion criteria were: available medical records with suspicion of partial ACL injury (history of knee sprain, positive Lachman test but often with a firm end-point, less than 5 mm differential laxity, and negative or grade I pivot shift test); no previous surgery in the same knee; MRI at 1.5 T with dedicated slides and specific enhancement of the pivot or at 3.0 T. Exclusion criteria were: recurrent tears; multiligamentous injury; inconsistent or inadequate MRI.

**Fig. 1:**
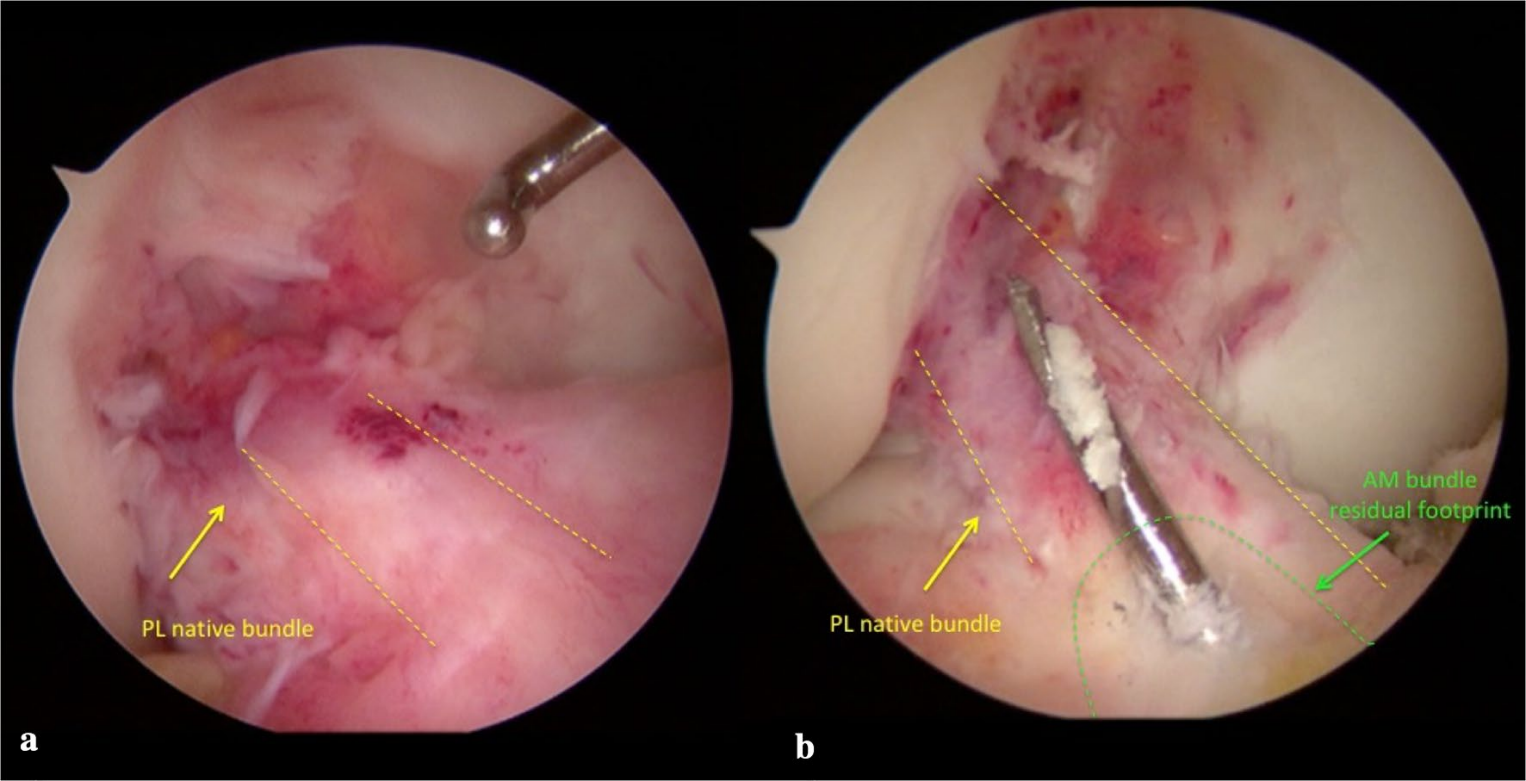
**a** Intraoperative arthroscopic view of isolated partial lesion of AM and **b** guidewire introduced in articular space form tibia in out–in fashion and placed on tibial remnant of AM bundle

Confirmation of partial ACL injury was obtained during arthroscopy using different intraoperative tests (Fig. 1a). First, direct evaluation was carried out by probing the tension of the residual safe bundle; intraoperative anterior drawer and Lachman tests were performed under direct visualization; finally, since AM bundle tears were found in almost all cases, PL bundle tension was evaluated by probing the knee in figure-of-4 (Cabot’s) position [35]. When all these signs proved to be positive, a partial tear was confirmed. Following these criteria, in 35 cases, we detected an isolated tear of the AM bundle, of which 23 were femoral avulsions while 12 could be described as tears of the AM bundle at its femoral side. Only one patient showed a single PL bundle tear.

### Surgical technique

**Fig. 2:**
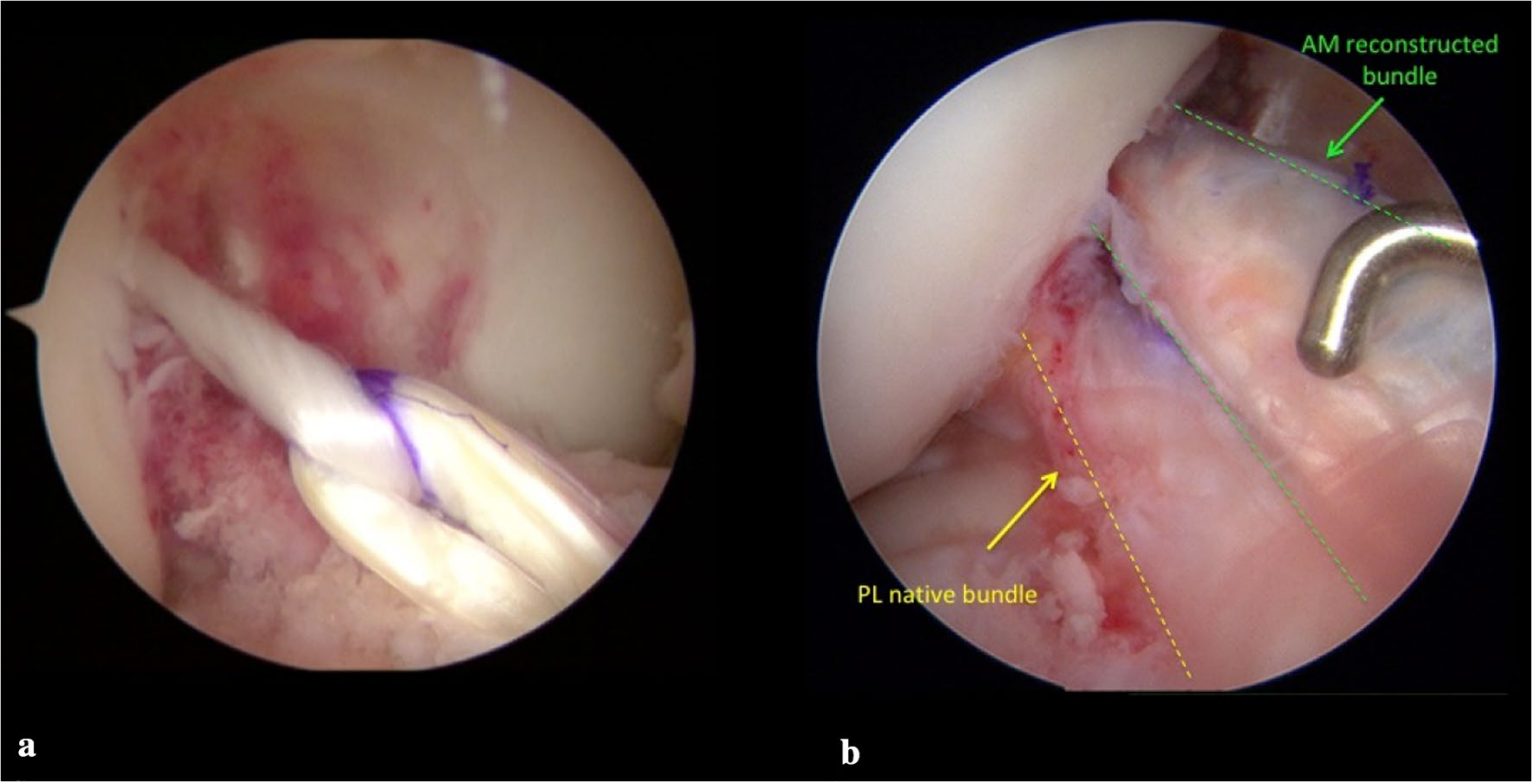
**a** Graft passage from tibial to femur side using Endobutton^®^ CL Ultra (Smith & Nephew, Warsaw, IN) and **b** final reconstruction of double bundle made by PL native bundle and AM autologous ST graft bundle. Evaluation of tension of final double bundle construct by probe

Isolated AM bundle reconstruction was performed in 35 patients, while 1 patient underwent substitution of the isolated PL bundle. All patients were operated using spinal anesthesia, antibiotic prophylaxis with 2 g cefazolin, and tourniquet. In all cases, the autologous duplicated semitendinosus (ST) tendon was used to reconstruct the torn bundle. The same surgeon performed a transtibial technique with creation of a femoral tunnel via in–out technique, after the positioning of a K-wire oriented in the same direction as the residual bundle and very close to it (Fig. 1b). In all patients, the fixation devices used were Endobutton^®^ CL Ultra (Smith & Nephew, Warsaw, IN) (Fig. 2a) and Biointrafix^®^ (DePuy Mitek, Raynam, MA) [7]. After positioning of the graft, direct visualization of the absence of a notch conflict and evaluation of tension were carried out with a probe (Fig. 2b). All intraoperative findings and postoperative complications were recorded.

### Physical functioning and quality of life

All patients underwent the same specific postoperative rehabilitation protocol. Follow-up visits were planned after 1, 3, 6, and 12 months and yearly until last follow-up. The minimum follow-up was considered to be 48 months. After 6 and 12 months and then yearly, all patients were evaluated using the International Knee Documentation Committee (IKDC) and the Knee Injury and Osteoarthritis Outcome Score (KOOS) [3, 30]. At the same intervals, knee laxity was measured using a KT2000^®^ arthrometer (MEDmetric Corp, San Diego, CA). Finally, subjective satisfaction level was calculated using the Short Form 12 (SF-12) questionnaire [38].

At 12-month follow-up, we simply asked patients to declare if they had returned to their preoperative sport level with a yes-or-no questionnaire—in the specific format where “return to sport” means return to the same level of competitive sport as previous to the injury. Moreover, sports functional recovery was coded as “yes”, able to play, and “no” if they had given up sport or had not been able to return to sport for reasons other than the knee—finally adding specifically when they returned to sport activity after surgery.

### Statistical analysis

Data were analyzed using IBM SPSS software version 23.0 for Windows (IBM Corporation, Armonk, NY). Statistical analysis was first performed based on an a priori assumption of *p* = 0.05 and calculation of variance to justify that the population from which it was extracted was generally homogeneous. All data were tested for normal distribution using the Kolmogorov–Smirnov test. Finally, Student’s *t* test was used both to compare preoperative and postoperative qualitative and quantitative laxity data and to evaluate subjective and objective IKDC and KOOS scores and SF-12 questionnaire. We performed multivariate linear regression analyses to determine which factors affected subjective and objective clinical results and functional recovery time. The independent variables in multivariate linear regression included demographic (sex, age, dominant affected side), preoperative subjective [subjective IKDC, KOOS tot., both SF-12 physical component summary (PCS) and SF-12 mental health composite scale (MCS)] and objective (objective IKDC, KT-2000) scores, and associated lesions (meniscal tears, chondral lesions).

## Results

All subjects completed the minimum follow-up of 48 months. The median follow-up was 64 months (48–84 months). No intra- or postoperative complications were recorded.

A single patient after 12 months referred a residual instability, maybe due to another knee sprain: he underwent reconstruction of the second bundle (considered safe in the index operation) at another facility.

Several associated lesions were found during arthroscopy: 12 medial meniscus and 3 lateral meniscus tears, 6 chondropathies of the medial femoral and tibial condyle, respectively, and 3 chondropathies of the lateral femoral condyle (6 grade 2 combined chondropathies of the femur condyle and medial tibial plateau, 2 grade 1 chondropathies of the lateral femur condyle, and 1 grade 1 of the lateral femur condyle, according to Outerbridge’s classification) [29]. All meniscal lesions were managed by debridement and regularization, while radiofrequency thermal shrinkage was performed for chondral alterations. In the preoperative setting, 57% of patients reported pain and 92% sensed giving way in their knee. The clinical examination showed a positive Lachman test in 62% of cases, with a soft endpoint. On the KT2000 evaluation, the preoperative mean differential laxity was 4.7 mm (1–12 mm; SD 2.04 mm) and 1.8 mm at final follow-up (0–4 mm; SD 1.08 mm) (*p* < 0.001). The subjective IKDC reported a mean value of 58.7 (42–91; SD 12.17) and 96.1 (86–100; SD 4.35) at the preoperative and final follow-up assessment, respectively (*p* < 0.001). Preoperatively, most patients were classified as C on the objective IKDC score (21 cases), while the remainder included 9 class D and 6 class B. At final follow-up, 55% of patients reported a IKDC score of grade A and 45% grade B (*p* < 0.001). The mean total KOOS score was 64.4 (46–86; SD 10.33) preoperatively and 96.8 (88–100; SD 3.77) at final follow-up (*p* < 0.001) (Table 2). Regarding the yes-or-no questionnaire submitted after 12 months of follow-up, 33 patients declared to have returned to their preinjury level of sport, whereas 3 patients had to change their activities. On the same form, they declared that the mean period they needed to get back to their preoperative level of sport activities was 5.1 months (4–7 months; SD 0.82 months). At final follow-up, we recorded high levels of satisfaction on SF-12 for both physical and mental scores [preop. PCS 31.2 (24–40; SD 4.08) and MCS 38.1 (30–46; SD 4.42), 48 months postop. PCS 52.2 (48–56; SD 2.65) and MCS 53.6 (48–60; SD 3.37), *p* < 0.001].

**Table 2:** Results

Score	KT2000	KOOS	IKDC				
			Subjective	Objective			
				A (%)	B (%)	C (%)	D (%)
Preoperative score	4.7 mm	64.4	58.7	0	17	58	25
Postoperative score	1.8 mm	96.8	96.0	55	45	0	0
*p*^a^	< 0.001	< 0.001	< 0.001				
Time to return to full physical activity 5.1 months							

Values expressed as median (range)

^a^*p* < 0.05 considered to indicate statistical significance

On multivariate analysis, we found poor correlation between the dependent and independent variables studied, meaning that we could not identify any predictors for the above-cited outcomes. Indeed, none of the demographic characteristics (sex, age, and dominant affected side) or preoperative subjective [subjective IKDC, KOOS tot., and SF-12(PCS and MCS)] and objective (objective IKDC and KT-2000) scores contributed to any of the subjective and objective clinical results, nor to time to return to sport (Tables 3, 4, 5). Moreover, regarding patient satisfaction (Table 3), medial meniscal tears and medial combined grade 2 femorotibial chondral lesions negatively correlated with patient satisfaction scores. Beyond that, we identified no other variables negatively related to the outcomes measured (Tables 3, 4, 5).

**Table 3:** Multivariate analysis of subjective clinical results

Factor	48-month follow-up subjective IKDC			48-month follow-up KOOS			48-month follow-up SF-12 PCS			48-month follow-up SF-12 MCS		
	*F*-stat	*p*-value	Negative effect	*F*-stat	*p*-value	Negative effect	*F*-stat	*p*-value	Negative effect	*F*-stat	*p*-value	Negative effect
Sex	1.2787	0.3566	None	1.4367	0.2443	None	2.6687	0.1453	None	1.6586	0.1335	None
Age	1.1323	0.4224	None	1.3346	0.5349	None	3.2253	0.3565	None	2.2253	0.2455	None
Dominant affected side	1.2395	0.7254	None	1.0974	0.6568	None	1.7988	0.4468	None	1.5478	0.3556	None
Preop. subjective IKDC	1.8102	0.9653	None	1.3577	0.6435	None	1.9975	0.8773	None	1.3585	0.6673	None
Preop. KOOS	0.1321	0.7531	None	0.3589	0.5598	None	1.5487	0.5598	None	0.2476	0.5336	None
Preop. SF-12	0.4681	0.1785	None	0.8572	0.2487	None	0.9682	0.4752	None	0.5378	0.2576	None
Preop. objective IKDC	1.3589	0.8657	None	1.5683	0.7689	None	1.3697	0.5883	None	0.9752	0.4225	None
Preop. KT-2000	1.0389	0.9653	None	1.3387	0.7592	None	1.4687	0.3557	None	0.9975	0.6659	None
Preop. meniscal tears	5.6741	0.7046	Medial meniscal tears	5.2582	0.5682	Medial meniscal tears	2.6684	0.6225	None	2.8966	0.4952	None
Preop. chondral lesions	4.3422	0.7652	Grade 2 combined medial femorotibial chondral lesions	5.1368	0.4697	Grade 2 combined medial femorotibial chondral lesions	3.2556	0.4558	None	2.9588	0.3685	None

**Table 4:** Multivariate analysis of objective clinical results

Factor	48-month follow-up objective IKDC			48-month follow-up KT-2000		
	*F*-stat	*p*-value	Negative effect	*F*-stat	*p*-value	Negative effect
Sex	0.7846	0.1224	None	0.8425	0.0935	None
Age	0.9975	0.1445	None	0.9935	0.1356	None
Dominant affected side	1.4565	0.2399	None	0.9655	0.0864	None
Preop. subjective IKDC	1.2377	0.6834	None	0.8774	0.5628	None
Preop. KOOS	0.2536	0.6358	None	0.9628	0.4233	None
Preop. SF-12	0.2463	0.1427	None	0.3476	0.1297	None
Preop. objective IKDC	0.9769	0.5344	None	1.1576	0.1885	None
Preop. KT-2000	1.3290	0.3501	None	1.4266	0.4289	None
Preop. meniscal tears	0.9732	0.5639	None	0.8772	0.2916	None
Preop. chondral lesions	1.1254	0.4209	None	0.9968	0.3298	None

**Table 5:** Multivariate analysis of functional recovery time

Factor	Time to return to sport		
	*F*-stat	*p*-value	Negative effect
Sex	0.7846	0.0828	None
Age	0.9975	0.0792	None
Dominant affected side	1.4565	0.1893	None
Preop. subjective IKDC	1.2377	0.1997	None
Preop. KOOS	0.2536	0.2917	None
Preop. SF-12	0.2463	0.0726	None
Preop. objective IKDC	0.9769	0.2718	None
Preop. KT-2000	1.3290	0.1892	None
Preop. meniscal tears	0.9732	0.0968	None
Preop. chondral lesions	1.1254	0.0955	None

## Discussion

In the present retrospective study, we decided to evaluate both clinical outcomes and the time needed to return to sport after selective surgical reconstruction of a single deficient ACL bundle in the context of partial ACL tear. The study was motivated by the absence of strong evidence supporting surgical augmentation or reconstruction, or even nonsurgical conservative rehabilitation protocols, as treatment for partial ACL tear [1, 22, 34]. Moreover, scientific literature provides little information about clinical outcomes of surgical management of such lesions [1, 5, 6, 16, 20, 33, 35]. To the best of our knowledge, this study is the first to introduce patients’ self-reported timing of return to sport after selective partial ACL reconstruction.

**Fig. 3:**
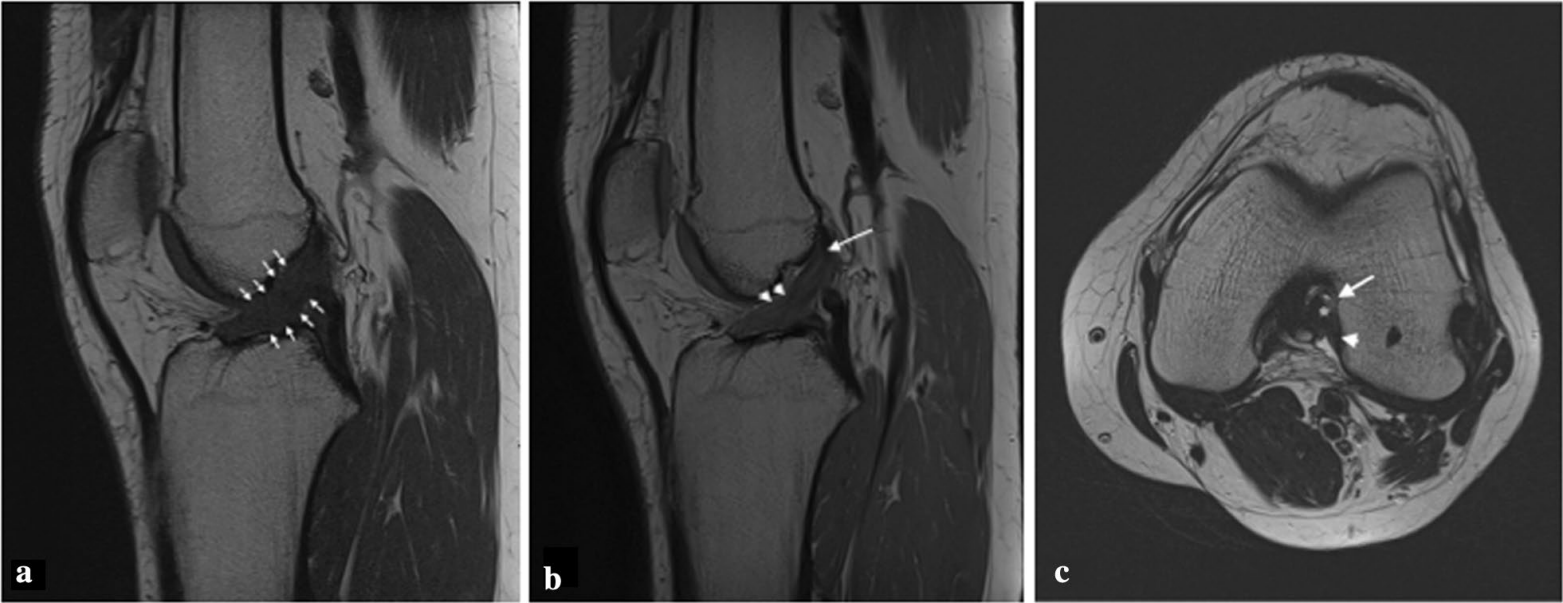
**a** Sagittal T1-weighted MR image revealing diffuse thickening, poor definition of anterior edge of ACL (arrows) with increased signal intensity within the ACL substance; **b** 3D PD-weighted, sagittal multiplanar reformatted (MPR) image showing abnormal intrasubstance high signal intensity of middle and distal third of ACL (arrowheads) and well-discernible fibers in proximal region (arrow); **c** axial T1-weighted MRI showing focal fluid signal along the midsubstance of ACL (arrow) in the intercondylar notch. Posterior fibers of ACL remain attached to femur (arrowhead), while anterior fibers seem to wear thin and detach (asterisk)

Despite analyzing a small sample of patients, which was in any case in line with literature, and considering the percentage of partial tears compared with total ACL tears treated at our institution (about 10–12%), our experience indicates that a partial tear may be suspected when the preoperative clinical assessment shows a combination of poor results on IKDC and KOOS scores, and a Lachman test with a delayed firm end-point associated with arthrometric laxity between 3 and 5 mm. Magnetic resonance imaging, the technique of choice to evaluate the status of the ACL, in some cases is not conclusive as there is an overlap in the appearance of a partial tear and a complete tear. A multiplanar approach and the use of high field strengths in 1.5-T and 3-T MRI systems are essential for accurate diagnosis of ACL injuries (Fig. 3a–c). The use of angled sagittal images or three-dimensional (3D) sequences obtained with thinner slices and multichannel phased-array coils can improve the accuracy of MR imaging for diagnosis of ACL tears. However, adequate MRI study may help in the final diagnosis [14], which is nevertheless ultimately achieved during arthroscopy, when dynamic tests and the perception of the surgeon regarding the good quality of the residual bundle confirm an isolated partial lesion. Regarding clinical outcomes, we found that subjective and objective results as well as the postoperative laxity assessment were good and similar to those reported in other studies [1, 6, 26]. Recently, Sonnery-Cottet and Colombet [36] published a review article analyzing outcomes after partial ACL reconstruction. Firstly, they identified only 17 studies and 3 metaanalyses with a total of 805 cases of partial ACL selective reconstruction. They also specified that only two studies analyzed more than 50 cases [27, 37]. Secondly, they found significant improvements in the subjective and objective scores in all examined studies, therefore justifying selective reconstruction in case of partial ACL tear. Moreover, to strengthen the utility of this procedure, Adachi et al. [1], who compared 40 selective reconstructions with a control group of conventional total reconstructions, found that preserving the intact bundle reduces postoperative knee laxity but mostly adds joint stability and proprioception thanks to the preservation of a greater amount of mechanoreceptors. Even Pujol et al. [28], in their case-control study, reported the same knee laxity results as above, but only for the first year after surgery, while the difference in laxity was not significant after 2 years. Focusing on postoperative knee laxity, only one recent study has compared results after selective reconstruction of ACL in patients with preoperative Kneelax arthrometer ≤ 5 mm and pivot shift test < II grade (group A) and in patients with Kneelax arthrometer ≥ 5 mm and/or pivot shift test ≥ II grade (group B) [23]. They found that partial reconstruction could significantly improve the stability and function of the affected knee. However, the group B patients still had anterior instability in the affected knee after partial reconstruction. Therefore, they may have required increased-diameter bundle or conventional total reconstruction. This study underlined the importance of evaluation of the residual bundle’s quality and the necessity of introducing other evaluation instruments other than the perception/feeling of a single surgeon in the decision-making process regarding whether to perform selective reconstruction of a single bundle. Furthermore, as reported above, many studies in literature have described the safety of partial ACL reconstruction along with clinical objective and subjective outcomes, but none of them determined or even analyzed the time to return to sport after this procedure. Moreover, none of them objectively defined the safe time to return to sport after partial ACL reconstruction. Albeit only with a simple yes-or-no questionnaire at 12 months of follow-up, we asked our patients whether they had returned to their preoperative level of sports, and in case of a positive answer, we asked them to write down the timing. We found that 33 of 36 patients declared that they returned to their preoperative level of sport at a mean time of 5.1 months (4–7 months). Only three patients at 12 months answered “no” and admitted to have changed their level of sport. At final follow-up, we found no new ruptures and only one patient (the one who had PL bundle selective reconstruction) referred a persistent instability. Regarding possible predictors of outcomes, we recorded a negative correlation between the presence of medial meniscal tears and medial grade 2 coupled femorotibial chondral lesions with the final subjective clinical outcomes, meaning that the more the lesion was associated with the medial compartment of the knee, the worse the satisfaction of the patient. Nevertheless, the same negative correlation was not found for the objective outcomes or for time to return to sports. Unfortunately, with a minimum 4-years follow-up, we cannot predict whether this negative correlation could mean a higher risk of failure or osteoarthritic degeneration of the knee joint in the future.

Our study has several limitations. First of all, it is a retrospective nonmulticentric study with a small patient sample. We did not evaluate knee instability using the pivot shift test, and we did not perform any MRI after surgery to evaluate the integration of the reconstructed graft. Moreover, the minimum of 4-year follow-up is rather short, even if new ruptures usually occur within the first year after surgery. Finally, the decision to perform selective bundle reconstruction was finally taken based on the arthroscopic assessment and was thus related to the surgeon’s experience. In fact, it was impossible to quantify the percentage of preservation of native ACL fibers, so the definition of a good residual bundle was only qualitative and was made by the feeling/perception of the surgeon during the arthroscopic tests described above. However, partial ACL ruptures are usually less frequent than complete or subtotal injuries, and the assessment of such lesions by a single experienced surgeon may be acceptable to ensure adequate interpretation of the results.

Despite the above-mentioned limitations, the quick recovery time reported in our study along with the good clinical results described in literature may justify selective reconstruction as a method to consider in the presence of partial ACL rupture. Specific technical and diagnostic steps should be performed to reveal a suspicion of partial ACL lesion, which nevertheless certainly needs to be confirmed by intraoperative arthroscopic evaluation if there is any suspicion of partial ACL tear.

## Data Availability

The dataset supporting the conclusions of this article is included within the article.

## Abbreviations

ACL: Anterior cruciate ligament
AM: Anteromedial
PL: Posterolateral
MRI: Magnetic resonance imaging
ST: Semitendinosus tendon
IKDC: International Knee Documentation Committee
KOOS: Knee Injury and Osteoarthritis Outcome Score
SF-12: Short Form 12
PCS: Physical component summary
MCS: Mental health composite scale
